# Early Dendritic Dystrophy in Human Brains With Primary Age-Related Tauopathy

**DOI:** 10.3389/fnagi.2020.596894

**Published:** 2020-12-07

**Authors:** Yan-Bin Shi, Tian Tu, Juan Jiang, Qi-Lei Zhang, Jia-Qi Ai, Aihua Pan, Jim Manavis, Ewen Tu, Xiao-Xin Yan

**Affiliations:** ^1^Medical Doctor Program, Xiangya School of Medicine, Central South University, Changsha, China; ^2^Department of Anatomy and Neurobiology, Xiangya School of Medicine, Central South University, Changsha, China; ^3^Faculty of Health and Medical Sciences, University of Adelaide, Adelaide, SA, Australia; ^4^Department of Neurology, Brain Hospital of Hunan Province, Changsha, China

**Keywords:** Alzheimer’s disease, brain aging, neurodegeneration, neuritic dystrophy, synaptic pathology

## Abstract

Dystrophic neurites (DNs) are found in many neurological conditions such as traumatic brain injury and age-related neurodegenerative diseases. In Alzheimer’s disease (AD) specifically, senile plaques containing silver-stained DNs were already described in the original literature defining this disease. These DNs could be both axonal and dendritic in origin, while axonal dystrophy relative to plaque formation has been more extensively studied. Here, we demonstrate an early occurrence of dendritic dystrophy in the hippocampal CA1 and subicular regions in human brains (*n* = 23) with primary age-related tauopathy (PART), with neurofibrillary tangle (NFT) burden ranging from Braak stages I to III in the absence of cerebral β-amyloid (Aβ) deposition. In Bielschowsky’s silver stain, segmented fusiform swellings on the apical dendrites of hippocampal and subicular pyramidal neurons were observed in all the cases, primarily over the stratum radiatum (s.r.). The numbers of silver-stained neuronal somata and dendritic swellings counted over CA1 to subiculum were positively correlated among the cases. Swollen dendritic processes were also detected in sections immunolabeled for phosphorylated tau (pTau) and sortilin. In aged and AD brains with both Aβ and pTau pathologies, silver- and immunolabeled dystrophic-like dendritic profiles occurred around and within individual neuritic plaques. These findings implicate that dendritic dystrophy can occur among hippocampal pyramidal neurons in human brains with PART. Therefore, as with the case of axonal dystrophy reported in literature, dendritic dystrophy can develop prior to Alzheimer-type plaque and tangle formation in the human brain.

## Introduction

Malformations of axonal or dendritic processes are generally referred to as neuritic pathology. These lesions can occur in the nervous system under various conditions, such as traumatic brain injury (Gentleman et al., 1993; Tom et al., 2004; Kelley et al., 2019), cerebral stroke (Carmichael et al., 2017), temporal lobe epilepsy (Judkins et al., 2006), and most age-related neurodegenerative diseases (Critchley, 1929; Dickson et al., 1994; Gai et al., 1995; Maat-Schieman et al., 1999). Specifically, the silver-stained senile plaques observed in the brains of non-demented and demented elderly over a century ago were shown to consist of some extracellular amorphous substance and swollen neuritic structures (Critchley, 1929; García-Marín et al., 2007; Ohry and Buda, 2015). The former was further found to be amylophilic (i.e., binding to such as Congo red and thioflavin) and related to the peptides named as β-amyloid (Aβ; Oifa, 1973; Glenner and Wong, 1984; Coria et al., 1988). Aβ antibody labeling confirmed the extracellular peptide accumulation at senile plaques and, additionally, as diffuse parenchymal plaques as well as vascular and meningeal amyloidosis (Allsop et al., 1986; Yamaguchi et al., 1988; Braak and Braak, 1991; Jellinger and Bancher, 1998). The plaque-associated neurites were called dystrophic neurites (DNs), as their precise origin from axons or dendrites was initially not clear about.

Follow-up electron microscopy (EM) and immunohistochemical studies advanced the characterization of plaque-associated DNs. Thus, at EM levels, these DNs appeared to be largely swollen axonal terminals containing synaptic vesicles (Luse and Smith, 1964; Cras et al., 1991; Masliah et al., 1991). Light microscopic immunohistochemical studies further revealed the accumulation of general and specific presynaptic and neurotransmitter proteins in the DNs, such as chromogranin-A, synaptophysin, choline acetyltransferase, tyrosine hydroxylase, glutamate decarboxylase, and vesicular glutamate transporters (Struble et al., 1982, 1987; Walker et al., 1985; Shoji et al., 1990; Brion et al., 1991; Munoz, 1991; Tourtellotte and Van Hoesen, 1991; Su et al., 1993, 1996, 1998; Grace et al., 2002; Fiala, 2007; Zhang et al., 2009). Notably, these axonal DNs co-expressed the Aβ-producing proteins, i.e., β-amyloid precursor protein (APP; Shoji et al., 1990; Cras et al., 1991), β-secretase 1 (BACE1; Zhang et al., 2009; Cai et al., 2010, 2012; Yan et al., 2014; Sadleir et al., 2016), and γ- and η-secretase components (Su et al., 1993; Willem et al., 2015), thereby likely serving as an important origin of the plaque-forming Aβ. The amyloidogenic DNs can be induced experimentally by various traumatic insults in wild-type animals, resulting in a certain degree of local Aβ accumulation (Gentleman et al., 1993; De Gasperi et al., 2012; Yan et al., 2012b; Deng et al., 2014), and in transgenic Alzheimer’s disease (AD) mouse models, accelerating amyloid plaque formation (Zhang et al., 2010; Yan et al., 2012a; Tajiri et al., 2013; Shishido et al., 2016).

Evidence also exists in support of the presence of non-axonal, likely dendritic, DNs around neuritic plaques. In fact, previous studies have classified two types of plaque-associated DNs, with type I containing phosphorylated tau (pTau)-positive paired helical filaments (PHF) and type II expressing chromogranin-A and APP (Brion et al., 1991; Munoz and Wang, 1992; Wang and Munoz, 1995; Su et al., 1996, 1998; Thal et al., 1998). Accordingly, the so-called type I neurites could be dendritic in theory. However, likely due to a down-regulated expression, the microtubule-associated protein-2 (MAP2), a typical dendritic marker, could not immunohistochemically visualize these putative dendritic DNs in human or transgenic AD mouse brains (Zhang et al., 2009; Cai et al., 2010; Takahashi et al., 2013).

The temporal or causal relationship between neuritic pathogenesis and Aβ accumulation is one of the important issues in understanding the onset and progression of senile plaque formation and AD pathogenesis. Axonal and dendritic neurites appear to undergo dynamic changes along with Aβ accumulation during plaque development (Grutzendler et al., 2007; Yan et al., 2014; Blazquez-Llorca et al., 2017). Axonal dystrophy can also occur in human and transgenic AD mouse brains prior to microscopic Aβ deposition (Stokin et al., 2005; Adalbert and Coleman, 2013). Dendritic alterations, especially spine loss, may be an early event in many neurodegenerative diseases including AD (Herms and Dorostkar, 2016), while it remains less clear whether microscopically overt dendritic dystrophy may occur in human brain prior to plaque and tangle formation. To address this question, postmortem human brains characteristic of primary age-related tauopathy (PART) were examined in the current study. Morphological and quantitative analyses were carried on silver- and immuno-labeled hippocampal CA1 and subicular pyramidal neurons, with particular attention paid to dendritic alterations. For comparison, dendritic pathology around established senile plaque was examined in temporal lobe sections that were originally prepared for our recent studies (Hu et al., 2017; Zhou et al., 2018; Xu et al., 2019; Tu et al., 2020).

## Materials and Methods

### Human Brain Samples

The use of postmortem human brains was approved by the Ethics Committee of Central South University Xiangya School of Medicine, in compliance with the Code of Ethics of the World Medical Association (Declaration of Helsinki). Postmortem brains were banked through the willed body donation program during the past several years. In general, the brains were collected within 24 h after death, except in occasional cases when the donation occurred in the winter season or the donor’s body was kept in a cold chamber after death. Brain banking did not include the cases that used a respiratory for life maintenance over 24 h. The postmortem brains were assessed for optimal histological integrity as well as plaque and tangle pathologies following a standard protocol proposed by the China Human Brain Banking Consortium (Yan et al., 2015; Qiu et al., 2019). Specifically, AD-type lesions (if present) were scored following an examination of the sections from various brain regions according to the National Institutes of Health guideline (Jellinger and Bancher, 1998; Braak et al., 2006; Montine et al., 2012). For the current study, the temporal lobe sections from a total of 23 brains characteristic of PART were selected for further histological processing and study of the labeled neuronal profiles. The Braak’s neurofibrillary tangle (NFT) scores of these brains ranged from I to III, i.e., with pTau-immunoreactive neuronal profiles present largely in the entorhinal and hippocampal subregions but rarely in the neocortex, while no β-amyloidosis was observed in the cerebrum and subcortical structures (Table 1). Other morphological data were obtained from cases with both Tau and Aβ pathologies or free of these lesions, which were available from our recent studies (Zhou et al., 2018; Xu et al., 2019; Tu et al., 2020).

**Table 1:** Demographic, clinical, and pathological profiles of the studied subjects.

Case number	Age (years)	Sex	Clinical diagnosis and cause of death	Postmortem delay (hours)	Braak NFT stages	Thal Aβ phases
1	59	F	Respiratory failure	6	I	0
2	62	F	Lung cancer	16	I	0
3	62	F	Multiple myeloma	4	I	0
4	68	F	Coronary heart disease	6	I	0
5	86	M	Hypertension	6	I	0
6	68	M	Renal failure	8	I	0
7	70	M	Hepatocholangiocarcinoma	48	I	0
8	65	M	Lung cancer	6	II	0
9	67	M	Multisystem failure	6	II	0
10	68	F	Astrocytoma	4.5	II	0
11	70	F	Pneumonia	18	II	0
12	71	M	Cerebral stroke	8	II	0
13	72	M	Pneumonia	26.5	II	0
14	77	M	Multisystem failure	12	II	0
15	78	F	Ovary cancer	4.5	II	0
16	78	M	Prostate cancer	16.5	II	0
17	70	F	Pneumonia	12	II	0
18	81	M	Cardiovascular failure	6	II	0
19	89	F	Multisystem failure	16	II	0
20	70	M	Cardiac stroke	5.3	III	0
21	72	M	Aortic aneurysm	6	III	0
22	89	M	Multisystem failure	4	III	0
23	81	F	kidney failure	7	III	0
24	80	F	Alzheimer’s disease (AD)	22	VI	5
25	80	F	AD	5.5	VI	5
26	81	F	AD	5.5	VI	5
27	89	M	AD	8	V	4
28	91	M	AD	12	VI	5
29	62	F	Lung cancer	16	0	0
30	65	M	Heart stroke	30.5	0	0
31	77	M	Colon cancer	26.5	0	0
32	78	M	Prostate cancer	16.5	0	0

### Tissue and Section Preparations

The methods for histological processing of postmortem human brains were detailed in our recent publications (Hu et al., 2017; Tu et al., 2020). Briefly, we obtained previously cut (frontally), cryoprotected (at −20°C) cryostat (40 μm thick) temporal lobe sections from the middle one-third segment of the hippocampus for silver staining and immunohistochemistry. For each type of labeling, four sections with equal distance (~1,000 μm) were obtained from each brain. The cryoprotected sections were first brought to room temperature and then rinsed with phosphate-buffered saline (PBS, 0.01 M, pH = 7.3) twice to remove the embedding medium. Sections from multiple cases (four to six) were subsequently stained under identical conditions in each run of the experiments.

### Gros’ Modified Bielschowsky’ Silver Stain

Sections maintained in PBS were brought into de-ionized water (DW) for a few minutes (min), sensitized in freshly prepared 20% silver nitrate for 20 min, placed in DW again, and held while preparing the ammoniacal silver solution. The ammoniacal silver solution was prepared by adding ammonia drop-wise into 20% silver nitrate, which was mixed vigorously until the precipitate dissolved and the solution became clear. The sections were then impregnated in the dark in ammoniacal silver solution for approximately 15 min, allowing the neuronal fiber bundles and the white matter to stain black and the background to appear tan. The sections were further processed in a developer (2.5 ml of citric acid, 2 ml of 40% formalin, one drop of nitric acid, and 95.5 ml of tap water), washed in DW, and fixed with sodium thiosulphate. Finally, the sections were allowed to air-dry, dehydrated with ascending ethanol solutions, cleared with xylene, and coverslipped with a mounting medium.

### Immunohistochemistry

Sets of sections from four to six brains were batch-processed immunohistochemically with four primary antibodies, respectively, including rabbit anti-pTau (1:5,000, T6819, Sigma–Aldrich, St. Louis, MO, USA; Cai et al., 2010, 2012), rabbit anti-sortilin intracellular C-terminal domain (1:2,000, ab16640, immunogenic peptide corresponding to a.a. 800–831 of human sortilin, ab16686, Abcam Trading Shanghai Company Limited Shanghai, China; Hu et al., 2017), monoclonal mouse anti-Aβ 6E10 (1:5,000, #39320, Signet Laboratories Inc., Dedham, MA, USA), and rabbit anti-BACE1 (Zhang et al., 2009; Cai et al., 2010). The sections used for 6E10 immunolabeling were pretreated additionally with formic acid for 1 h at room temperature before the immunolabeling steps. All the sections were treated free-floating with 5% H_2_O_2_ in PBS for 30 min and 5% normal horse serum in PBS with 0.3% Triton X-100 for 1 h, followed by incubations with the above-mentioned primary antibodies at 4°C overnight. The sections were then reacted with biotinylated horse anti-mouse, rabbit, and goat IgGs at 1:400 for 1 h and the avidin–biotin complex reagents (1:400; Vector Laboratories, Burlingame, CA, USA) for another hour, with the immunoreactivity visualized in 0.003% H_2_O_2_ and 0.05% 3,3′-diaminobenzidine. The sections were coverslipped as described above in the silver-stained preparation.

### Imaging, Data Analysis, and Figure Preparation

Silver- and immuno-labeled sections were examined correlatively on an Olympus BX51 microscope (CellSens Standard, Olympus Corporation, Japan) to verify the case being characteristic of PART and to determine the stage of Braak NFT pathology. The sections were also scanned and imaged using the ×20 objective on a Motic-Olympus microscope equipped with an automated stage and imaging system (Wuhan, Hubei, China), which could yield a final auto-focused, montaged, and magnification-adjustable image covering the entire area of a glass slide. Low- (×2) and high- (×10 and ×20) magnification images over the area of interest (AOI) were exported from the Motic images for figure preparation. The Bielschowsky silver-stained Motic images were also used for quantitative analysis. Thus, the silver-stained pyramidal neurons (somata) and dendritic swellings were tagged using separate tools while scanning over the entire area from CA1 and the subiculum in each brain section, with the number of profiles counted afterwards. After obtaining the numbers from three equally distant temporal lobe sections, the means of labeled somata and dendritic swellings were calculated and expressed as the number of somatic and dendritic profiles per section, respectively. The data were recorded accordingly for individual brain cases, along with the information of Braak NFT staging. Finally, the numbers of silver-stained somata and dendritic swellings were entered into a Prism spreadsheet with the cases arranged in three groups, i.e., Braak NFT stages I, II, and III, respectively (GraphPad Prism 5.1, San Diego, CA, USA). The median numbers of labeled somata and dendritic swellings in the three groups were compared statistically with the nonparametric Kruskal–Wallis method, with *post hoc* tests performed to detect intergroup differences, if any. The numbers of somata and dendritic swellings among individual cases were plotted graphically, with Pearson correlation test carried out between the two sets of measurements. The minimal significant level of difference was set at *P* < 0.05. Figures were assembled with Photoshop 7.1.

## Results

### Braak Staging of Brain Cases With Primary Age-Related Tauopathy

**Figure 1:**
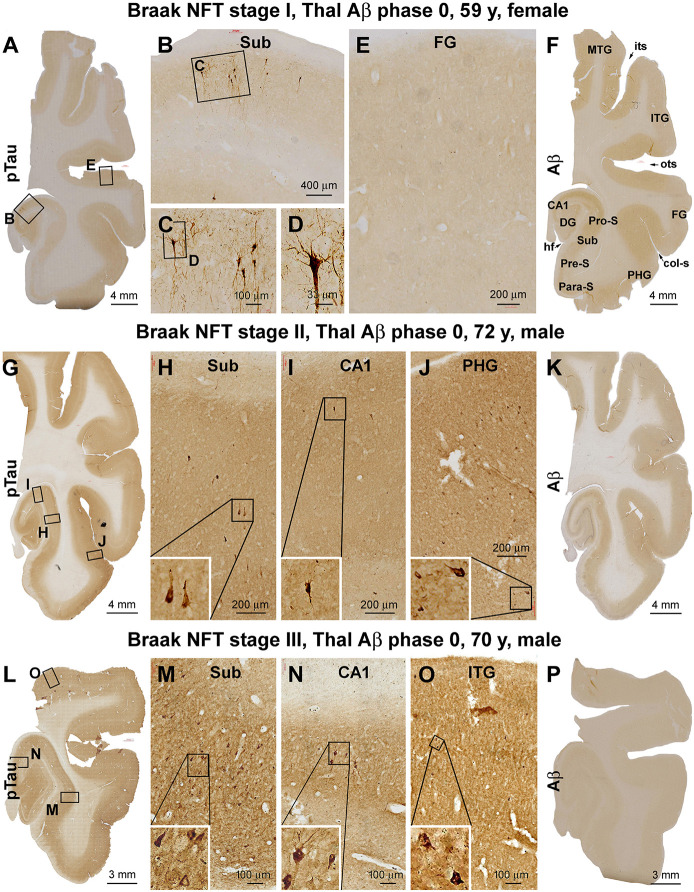
Verification and Braak staging of primary age-related tauopathy (PART) in postmortem human brains. Shown are micrographs of adjacent temporal lobe sections immunolabeled for phosphorylated tau (pTau) and β-amyloid (Aβ) from representative cases examined in the current study, with the patient’s biometrics (age and sex) and image panel arrangements indicated. The left and the middle panels show the regional distribution and morphology of pTau-immunoreactive neuronal profiles in the three cases with Braak stages I **(A–E)**, II **(G–J)**, and III **(L–O)** neurofibrillary tangle (NFT) lesions, respectively. The right panels **(F,K,P)** show the lack of Aβ deposition in the sections immunolabeled with the monoclonal antibody 6E10 in all the cases (scored as Aβ Thal phase 0). In the first case **(A–E)**, a small population of pyramidal neurons located around the border of CA1 and subiculum exhibited strong pTau immunoreactivity in the somata and dendritic arbors, with light labeling seen in their axons **(A–C)**, while no labeling is seen in the temporal neocortex (TC; **E**). In the second case **(G–J)**, pTau-labeled neuronal somata and processes occur in the CA1, transentorhinal, and entorhinal areas **(G–I)** and occasionally in the basal TC **(J)**. In the third case **(L–O)**, pTau-labeled neuronal somata and processes are densely present in the limbic areas **(L–N)** and also frequently seen over the temporal neocortical gyri **(O)**. col-S, collateral sulcus; DG, dentate gyrus; FG, fusiform gyrus; HF, hippocampal fissure; ITG, inferior temporal gyrus; MTG, middle temporal gyrus; its, inferior temporal sulcus; ots, occipito-temporal sulcus; PHG, parahippocampal gyrus; Sub, subiculum; Para-S, parasubiculum; Pre-S, presubiculum; Pro-S, prosubiculum. Scale bars and enlargements of local areas are as marked in the panels.

Based on the initial assessment of Aβ and pTau pathologies following brain banking, a total of 23 brain cases with PART were selected. The Braak NFT stages of the cases were verified according to the distribution of silver-stained and pTau-immunolabeled neuronal profiles (Braak and Braak, 1991; Braak and Del Tredici, 2020). Thus, there were seven cases that showed Braak stage I lesion, with pTau-immunolabeled pyramidal neurons localized to the transentorhinal area (Figures 1A–E). There were 12 cases that exhibited Braak stage II neuropathology, with pTau-labeled pyramidal neurons additionally present in the hippocampal CA1 sector, with a few neurons occasionally seen in the parahippocampal gyrus (PHG) at high magnification (Figures 1G–J). The remaining four cases were classified as having Braak stage III neuropathology, in which the pTau-positive neurons were also observed across the entorhinal cortical regions, while a few individually labeled neurons could be identified in the inferior temporal gyrus (ITG) by closer examination (Figures 1L–O). None of these brain cases exhibited Aβ deposition in the cerebral parenchyma, around the meninge, or at vasculature (Figures 1F,K,P; Table 1).

### Identification and Quantification of Dendritic Dystrophy in Silver-Stained Preparations

**Figure 2:**
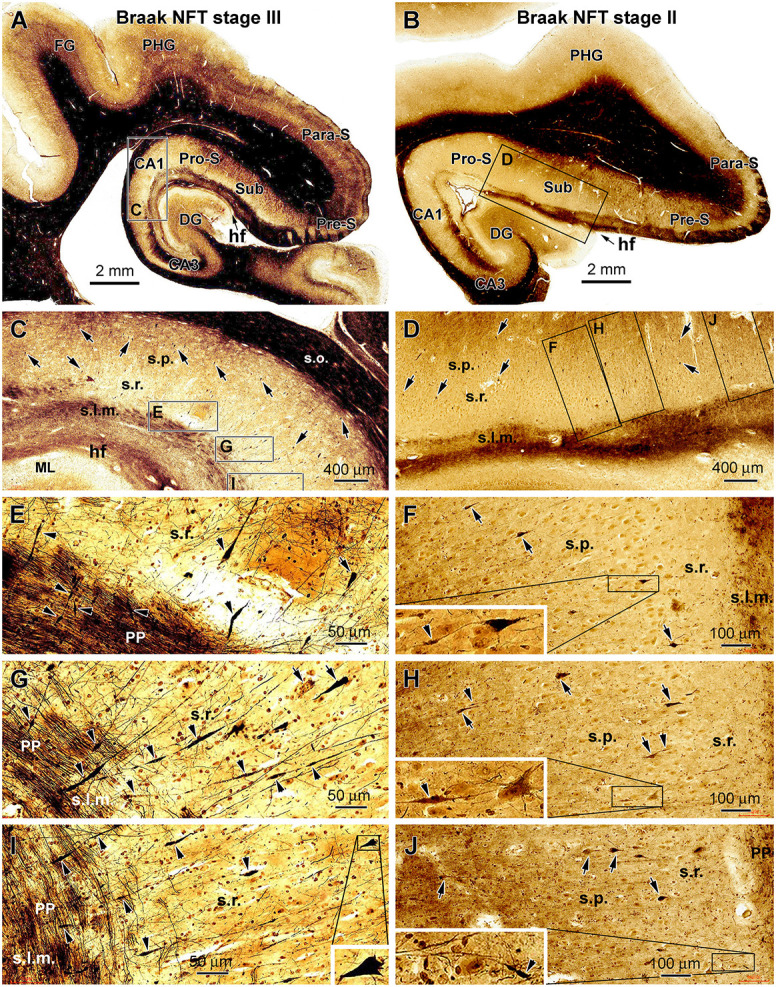
Representative silver-stained micrographs illustrating dystrophic dendritic profiles in the hippocampal formation in the brains with PART. Shown are low- and high-magnification views of the labeling in the temporal lobe sections from two cases (cases #22 and #16 in Table 1) with tauopathy at Braak stages III **(A,C,E,G** and **I)** and II **(B,D,F,H** and **J)**, respectively. The framed areas in the panels are enlarged as indicated. The arrows point to examples of silver-stained somata of CA1 and subicular pyramidal neurons, while the arrowheads point to examples of silver-stained dendritic swellings. The dystrophic dendritic parts are featured by segmented fusiform expansion along the course of the labeled dendritic processes, which are mostly located over the stratum radiatum (s.r.). Some dendritic swellings occur in the stratum lacunosum-moleculare (s.l.m.), intersecting with the fine axons of the perforant pathway **(C–J)**. In some cases, one silver-stained apical dendrite has repeated swellings along its proximal to distal continuation **(G,I)**. The swelling can be also seen at the proximal segments of dendrites **(F,H)**. Other abbreviations are as defined in Figure 1, and scale bars are as indicated.

Silver-impregnated neuronal profiles were found in all the cases examined in the current study, and they were present in the temporal lobe structures with a regional distribution consistent with that of pTau-immunolabeled profiles in the same brain. In the hippocampal formation, the neuronal profiles were localized to the subicular areas, CA1, or additionally the entorhinal cortex, depending on the Braak stages of the cases. In general, the labeled neuronal profiles were sparsely or individually distributed in the above-mentioned subregions. Consistent with the morphological features of hippocampal and subicular pyramidal neurons, the somata of the stained cells were conic or triangular in shape, localized to the stratum pyramidale (s.p.), and with their basal and apical poles facing the stratum oriens (s.o.) and stratum lacunosum-moleculare (s.l.m.), respectively. The basal and apical dendrites of these pyramidal neurons were often stained. Specifically, some of the apical dendrites were distinctly displayed and extended over long distances up to several hundreds of microns across the stratum radiatum (s.r.) and entering the s.l.m. (Figures 2C,E,G,I).

Segmental swelling or thickening was observed on the silver-stained dendritic processes of the hippocampal and subicular pyramidal neurons, which were most frequently seen in the s.r. and less frequently observed in the s.l.m. (Figures 2A–D). These labeled dendritic processes were largely oriented perpendicular to the hippocampal fissure (HF), some of which were connected proximally to the apical pole of a silver-stained pyramidal neuron. In the s.l.m., the swollen dendritic processes were intersected with silver-stained fine axonal fibers of perforant pathway, which runs in parallel with the hf. The swollen dendritic segments showed a fusiform appearance; each could extend from several to more than 50 μm in length. The swellings sometimes occurred repeatedly on the same dendrite from its proximal to distal extension. Regarding the labeling intensity, the swollen dendritic portions often exhibited heavy and solid silver impregnation, although those with lighter staining and of granular appearance were also observed (Figures 2E–J).

**Figure 3:**
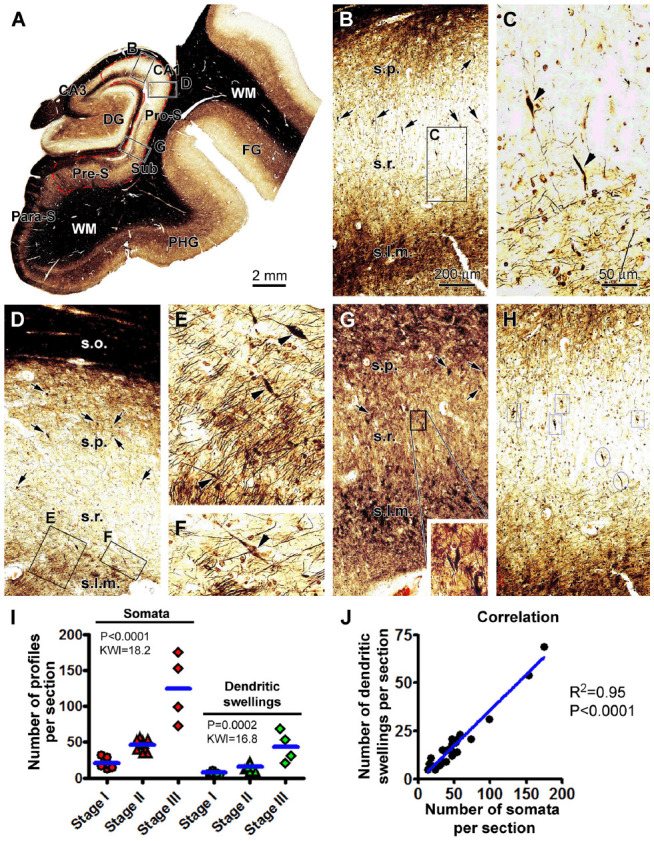
Quantification of silver-stained neuronal somata and dystrophic dendrites of the hippocampal CA1 and subicular pyramidal neurons relative to the Braak stages in the PART cases. **(A)** Case #12 in Table 1: low-magnification micrograph illustrating the subregions of the hippocampal formation, with the region marked with the red broken line defined as the area of interest (AOI) for the correlated quantification, including the CA1 and the subicular regions surrounding the dentate gyrus (DG). The framed areas in the panels are enlarged as indicated to illustrate the closer views of labeled neuronal somata and dendritic swellings **(B–G)**. **(H)** Portion of a screen print image to illustrate the taggling of silver-stained somata (framed) and dendritic swellings (circled) in the Motic scan imaging interface. **(I)** Numbers of silver-stained somata and dendritic swellings obtained within the AOI among the individual cases grouped based on the Braak stages. **(J)** Graph of Pearson correlation analysis. The results of the statistical test (Kruskal–Wallis nonparametric analysis) and correlation analysis are provided in these graphs. The abbreviations are as defined in Figures 1, 2. Scale bars are as indicated.

We carried out correlated quantitative analysis on the silver-stained neuronal somata and the dendritic swellings in individual brain cases (Figure 3). The somata and dendritic swellings in the same area in each section covering CA1 sector and subicular region were counted, with three equally distant sections per brain used to obtain an average number for a given case. Thus, while scanning across the above-mentioned region at low to high magnifications (×10 to ×20), silver-stained neuronal somata in the s.p. and dendritic swellings in the s.p., s.r., and s.l.m. were digitally tagged and then counted (Figures 3A–H). The numbers from the three sections were summed up, and the means of silver-stained pyramidal somata and dendritic swellings, expressed as the number of profiles per section, were calculated for a given brain. The means of all the cases studied were re-grouped according to the Braak stages (I, II, and III) of the brains.

The mean value of silver-stained neuronal somata was 21 ± 7.2 (mean ± S.D., same format below) per section in the group with Braak stage I tauopathy, 46 ± 7.9 per section in the group with Braak stage II lesions, and 125 ± 47.1 per section in the group with Braak stage III lesions. Based on the nonparametric Kruskal–Wallis (KW) analysis, there was a trend of increase in the medians of the labeled somata (*P* < 0.0001, KW index = 18.2), with *post hoc* test indicating a statistically significant difference for the median of the Braak stage I group relative to the stages II and III groups, respectively. The mean numbers of dendritic swellings were 7 ± 2.1, 16 ± 4.61, and 44 ± 21.8 per section for the groups with Braak stages I, II, and III lesions, respectively (Figure 3I). The median values of the dendritic swellings also showed a significant increase with the advance of the Braak stages (*P* = 0.0002, KW index = 16.8; Figure 3I). *Post hoc* tests indicated that the difference existed for the group with Braak stage I relative to the groups with stages II and III lesions (Figure 3I). We further carried out a correlative analysis (Pearson test) between the numbers of the silver-stained somata and dendritic swellings among the individual cases, which indicated that the two sets of numbers were positively correlated among the cases (*P* < 0.0001, *R*^2^ = 0.95; Figure 3J).

### Identification of Dendritic Dystrophy in pTau and Sortilin-Immunolabeled Sections

We reasoned that the dendritic dystrophy observed in the silver stain should be also detectable in immunohistochemical preparations with selected neuronal markers. pTau immunolabeling can often visualize the dendritic arbors of neurons at pre-tangle or early-tangle stages of lesions (Braak and Braak, 1991). Recently, the immunolabeling of sortilin, a type I membrane glycoprotein in the vacuolar protein sorting 10 protein (Vps10p) family of sorting receptors, has been found to mark the dendritic processes of the hippocampal and subicular pyramidal neurons (Hu et al., 2017; Xu et al., 2019). Therefore, we used pTau and sortilin immunolabeling to cross-validate the existence of dendritic dystrophy.

**Figure 4:**
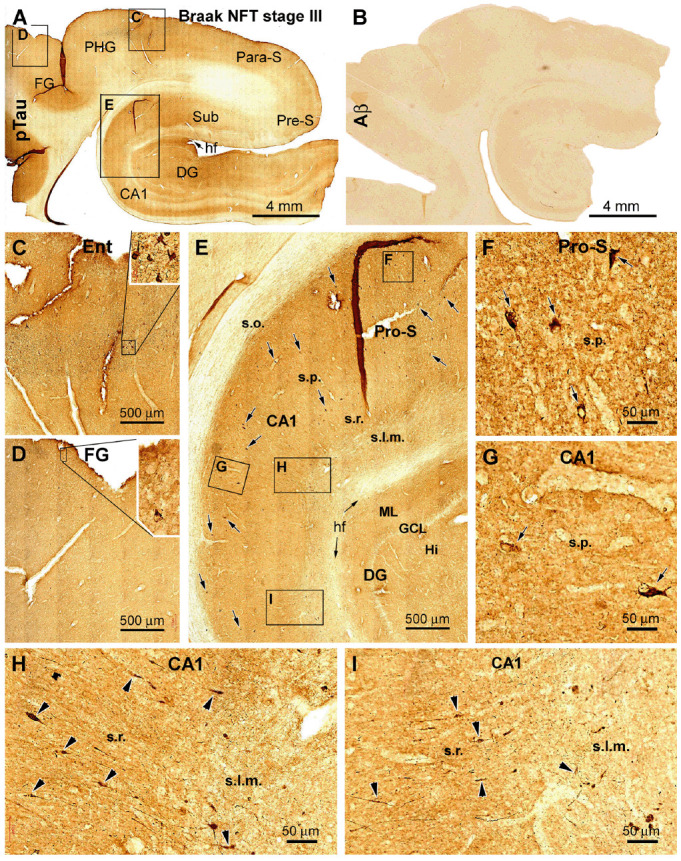
Cross-validation of the existence of dendritic dystrophy in the hippocampal formation with phosphorylated tau (pTau) immunohistochemistry. **(A)** Case #22 in Table 1: low-magnification view of pTau labeling over the hippocampal formation, the entorhinal cortex, and part of the TC. The boxed areas **(C–E)** are enlarged to show the distribution of pTau-immunolabeled neurons in the hippocampal formation **(E)** and entorhinal cortex **(C)** and occasionally in the neocortical fusiform gyrus **(D)**. **(B)** Lack of cerebral β-amyloid (Aβ) deposition in this case. The framed areas in **(E)** are further enlarged **(F–I)** to illustrate the somata (as pointed by arrows) of immunoreactive hippocampal and subicular pyramidal neurons. **(H,I)** Segmental fusiform swellings (as pointed by arrowheads) of the dendritic processes in the s.r. and stratum lacunosum-moleculare (s.l.m.). Additional abbreviations are as defined in Figures 1, 2. Scale bars in individual panels are as indicated.

Among the PART cases, especially those with Braak stages II and III lesions, pTau-immunoreactive dendritic swellings were found in the CA1 and subicular subregions, in addition to the labeled neuronal somata. Shown as an example, in the Motic-scanned sections from a Braak stage III case, pTau-immunoreactive neuronal somata were observed in the hippocampus proper and subicular areas (Figures 4A–I). They were present in the s.p. and morphologically characteristic of pyramidal neurons (Figures 4E–I). Fusiform expansions could be identified on the immunolabeled dendritic processes in the s.r. and less frequently in the s.l.m. These processes were oriented perpendicularly to the hf (Figures 4E,H,I). It should be noted that the pTau-immunoreactive dendritic swellings were not as distinctly displayed as those seen in the silver stain preparation because there existed a relatively high background reactivity, which is likely related to the expression of pTau in axonal elements. Therefore, we did not carry out quantitative analysis on pTau-labeled dendritic swellings.

**Figure 5:**
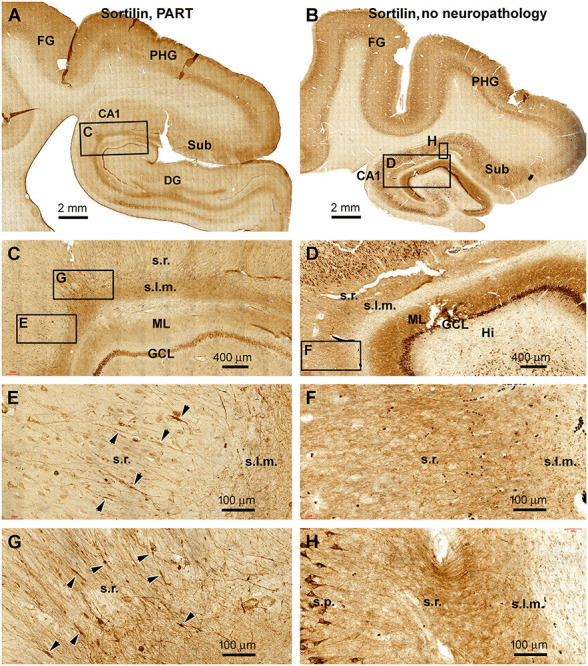
Observation of dendritic dystrophy in hippocampal formation in primary age-related tauopathy (PART) case in sortilin immunohistochemistry. **(A)** Sortilin labeling in the section adjacent to the one shown as Figure 4A (case #22 in Table 1). **(B)** Labeling in the comparable region in a case without cerebral pTau or Aβ pathologies (case #32). The framed areas are enlarged as indicated. In the PART case **(C,E,G)**, fusiform swellings (as pointed by arrowheads) are seen on the immunolabeled dendritic processes in the s.r. In contrast, no swelling is found on the dendritic processes in the control case **(D,F,H)**. Other abbreviations are as defined in Figure 1. Scale bars are as indicated.

In sections immunolabeled with the C-terminal sortilin antibody, the somata and dendritic processes of hippocampal, subicular, and cortical pyramidal neurons were well displayed in all the PART cases. This somal and dendritic labeling was also distinct in brain cases without amyloid and tau pathologies, which were used as assay control samples (Figures 5A–D; Table 1). Notably, in the PART cases, fusiform swellings could be identified on the dendritic processes of pyramidal neurons in the s.r. and s.l.m. in both the CA1 and subicular areas (Figures 5C,E,G). On the contrary, in the sections from control cases, no swollen dendritic processes could be found in the comparable anatomical locations (Figures 5D,F,H). Because there was an abundant immunolabeling of neurons and their processes in the sections, we did not quantify the dendritic swellings due to the same methodological concern as noted above in pTau preparations.

### Identification of Senile Plaque-Associated Dystrophic Neurites in AD Brains

For a morphological comparison with the dystrophic dendrites described in the PART cases, we were particularly interested in identifying putative dendritic DNs around established senile plaques. As denoted in the “Introduction” section swollen and tortuous presynaptic terminals are the main type of plaque-associated DNs, which can be distinctly visualized with BACE1 immunohistochemistry (Zhang et al., 2009; Cai et al., 2010; Takahashi et al., 2013; Blazquez-Llorca et al., 2017). On the other hand, the so-called type I DNs exhibiting strong pTau immunolabeling appear less likely axonal, given that they do not colocalize with chromogranin-A, APP, and BACE1 (Brion et al., 1991; Wang and Munoz, 1995; Su et al., 1996, 1998; Thal et al., 1998; Cai et al., 2010).

**Figure 6:**
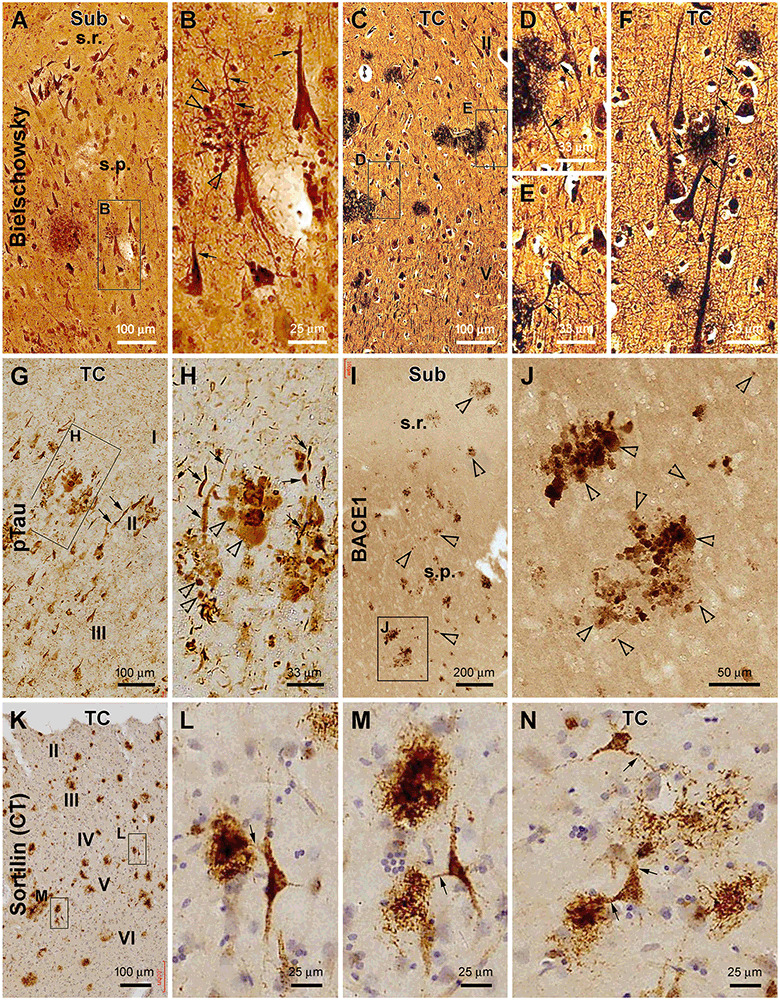
Characterization of plaque-associated axonal and dendritic pathologies. (**A–F**, cases #27 and 28 in Table 1) Silver-stained neuritic plaques in the subiculum and TC as indicated. (**G–J**, case #27) Phosphorylated tau- and β-secretase 1-immunoreactive neuritic profiles. (**K–N**, case #25) Neuronal profiles and extracellular plaques in the TC immunolabeled with the sortilin C-terminal antibody. Axonal dystrophic neurites (DN; as pointed by open arrowheads) appear as swollen globular profiles which occur in clusters and as small isolated sphericles as well. Dendritic neurites (as pointed by arrows) are elongated processes with local swelling or thinning, some of which are connected to the labeled neuronal somata. Panel arrangement, lamination, and scale bars are as indicated. I–IV, cortical layers. Additional abbreviations are as defined in Figures 1, 2.

In Bielschowsky’s silver stain preparation, putative axonal and dendritic DNs could be identified around senile plaques (Figures 6A–F). Thus, in small plaques wherein the amorphous silver stain was not evident, swollen globular profiles likely representing axonal DNs could occur near the apical dendrites of hippocampal pyramidal neurons. These apical dendrites and their branches became thinned while they intersected with the spherical axonal profiles (Figures 6A,B). The silver-stained somata of pyramidal neurons near the small plaques appeared to be tangled, given their uneven silver impregnation and distorted morphology (Figure 6B). Established senile plaques contained apparent amorphous (darkly stained) silver impregnation of putative amyloid deposition. The dendritic processes of nearby silver-stained neurons could be traced directly into these plaques and became thinner within the area occupied by the plaques (Figures 6C–F).

In pTau immunolabeling, we actually could differentiate two types of abnormal neuritic profiles around the senile plaques (based on the arrangement of neuritic clusters; Figures 6G,H). One type of neurites appeared spherical with varying size and staining intensity (Figure 6H, pointed by open arrows). These swollen neuritic profiles were morphologically identical to the presynaptic DNs labeled by BACE1 (Figures 6I,J), as also characterized previously (Zhang et al., 2009; Cai et al., 2010; Tu et al., 2020). Another type of pTau-positive neurites were elongated and thick neuronal processes exhibiting heavy immunoreactivity (Figures 6G,H). Some of them were apparently a portion or parts of the apical dendrites of pyramidal neurons, given their conic or triangular shape. Other abnormal dendritic processes could be traced over a long distance and had segmented thickening and thinning and sudden disruption in continuity or showed an uneven fibrillary contour (Figure 6H, pointed by arrows). This latter type appeared to be tangled dendritic neurites around the senile plaques based on their morphological features.

Sortilin is strongly expressed in neuronal somata and dendrites in the adult human brain, including in the dendritic spines as distinctly seen in the thorny excrescences of CA3 pyramidal neurons and hilar mossy cells (Xu et al., 2019). *Sortilin* C-terminal *fra*gments, named as sorfra in our recent study (Tu et al., 2020), can deposit as extracellular plaques in aged and AD human cerebrum (Hu et al., 2017; Tu et al., 2020). In the current study, we paid particular attention to the dendritic processes around sorfra plaques in sections immunolabeled with the C-terminal sortilin antibody. Dendritic branches were seen to connect with the sorfra plaques which consisted of either densely or loosely packed fibrillary deposits (Figures 6K–N). The dendritic processes appeared to break down and became fragmented as they run into the sorfra plaques (Figure 6N), suggestive of a dendritic contribution to the plaque-forming sorfra products. These sortilin-labeled dendritic processes rarely exhibited a tangle-like appearance as seen in pTau immunolabeling (Figures 6L–N).

## Discussion

PART is a recently defined neuropathological term based on autopsy examination of the brains from elderly individuals (Crary et al., 2014; Tsartsalis et al., 2018; Bell et al., 2019; Das et al., 2019). This lesion refers to the occurrence of tauopathy in the absence of Aβ deposition. The tauopathy is present primarily in the temporal lobe structures, with its extent ranging from Braak NFT stages I to IV (Kaufman et al., 2018; Kim et al., 2019; Wang et al., 2020). Individuals with PART are cognitively normal to their ages or have mild amnestic symptoms, while a minority could be severely demented (Besser et al., 2017; Jefferson-George et al., 2017; Quintas-Neves et al., 2019; Teylan et al., 2020). Whether PART represents a distinct clinicopathological entity or an early phase of AD progression remains a topic of discussion (Duyckaerts et al., 2015; Jellinger, 2019; Weigand et al., 2020). It should be noted that a recent study shows that tauopathy occurs frequently in subcortical regions even in cases with Braak stages I/II lesions, including the substantia nigra, inferior colliculus, locus coeruleus, and medulla oblongata in the brainstem, the caudate, putamen, and nucleus globus pallidus in the striatum, the hypothalamus, thalamus, and subthalamus in the diencephalon, and the cervical spinal cord. Moreover, the regional distribution and severity of the subcortical tauopathies in PART and AD cases are positively correlated with NFT Braak stages (Zhu et al., 2019). These data suggest a pattern of progression of tau pathogenesis with the advance of PART and AD.

The present study examined postmortem human brains obtained *via* a body donation program. Among the brains we have preserved during the past several years, a substantial proportion (approximately 60%) from individuals aged 65 and above (*n* = 61 out of 117 whole brains so far) showed neuropathological characteristics of PART. Other brains from the elderly donors exhibited AD-type neuropathology with Thal Aβ phases 1–6 and Braak NFT stages III and VI. The PART cases examined in the current study were 59–89 years old at death, 13 being male and 10 female, and died of various diseases (Table 1). Therefore, in agreement with previous reports (Crary et al., 2014; Kaufman et al., 2018; Kim et al., 2019; Wang et al., 2020), PART is commonly present in the brains of elderly Chinese obtained *via* community-based brain banking.

Synaptic degeneration is considered to best correlate with cognitive decline during brain aging and in AD (Scheff et al., 1990, 2001, 2007; Serrano-Pozo et al., 2011; Chen et al., 2018; Zhang et al., 2018; Cardozo et al., 2019). The loss of synapses in the brain is a “negative lesion” that requires quantitative assessment in regions affected in AD relative to the age-matched control (Scheff et al., 1990, 2001; Masliah et al., 1991; Kay et al., 2013). Recently, novel imaging and biofluid markers are developed for the assessment of synaptic degeneration in AD relevant to cognitive decline (Galasko et al., 2019; Lleó et al., 2019; Colom-Cadena et al., 2020; Mecca et al., 2020). The general trend of synaptic loss is associated with compensatory or reactive changes of some presynaptic axon terminals, which include axonal dystrophy characterized by globular swelling and aberrant sprouting, detectable microscopically as a positive neuropathology (Gonatas et al., 1967; Scheff and Price, 2003). The dystrophic presynaptic axon terminals appear to play an early and active role in neuritic plaque development as they produce Aβ (Fiala, 2007; Zhang et al., 2009; Yan et al., 2014). They can also develop prior to Aβ deposition in the human brain (Stokin et al., 2005). In this regard, it is important to explore whether the postsynaptic elements, specifically dendritic processes, could also undergo overt morphological changes before plaque formation.

Brains with PART are particularly suited to address the above-mentioned question because of the lack of Aβ deposition. We used a silver stain method to analyze pathological neuronal profiles, which can visualize subpopulations of neuronal somata along with their processes in an “all or none” manner (Toth et al., 1998). The CA1 and subicular pyramidal neurons are anatomically organized such that their apical dendrites and branches could be reliably identified over a fairly wide lamina from the s.r. to the s.l.m. We observed segmented fusiform swellings on the apical dendritic processes of the CA1 and subicular pyramidal neurons in the cases with PART in silver stain preparation. The numbers of silver-stained dendritic swellings and pyramidal neuronal somata measured over CA1 and the subicular regions were positively correlated among the individual cases, indicating an interdependent relationship between the two measurements. This same dendritic pathology was cross-validated in sections immunolabeled for pTau and sortilin.

We further confirmed the presence of dendritic, in addition to axonal, DNs in the vicinity of neuritic plaques in silver-stained and immunolabeled sections from AD brains. As well-established, swollen axonal DNs arranged as rosette-like clusters were visualized in silver, pTau, and BACE1 labeling, while isolated small sphericles were also seen between the clusters. Importantly, deformed dendritic processes coexisted around the dystrophic axonal clusters. These plaque-associated dendritic neurites exhibited more complex morphological alterations than the fusiform swelling seen in the PART cases, including segmental thickening, thinning, curvature, and disruption in continuity, and the tangle-like appearance indicative of dystrophic as well as degenerative changes. In sortilin C-terminal antibody labeling, dendritic processes connected to local neuronal somata extended towards and into sorfra plaques. The distal parts of the dendritic processes appeared to be dissolved and become a part of the deposited fibrils. Taken together, these findings support a notion that both axonal and dendritic dystrophy are involved in the formation of senile plaques in the human cerebrum, including providing the plaque-forming Aβ and sorfra (Knowles et al., 1999; Tsai et al., 2004; Spires et al., 2005; Fiala, 2007; Yan et al., 2014; Hu et al., 2017).

The mechanism underlying the early occurrence of dendritic dystrophy in hippocampal and subicular pyramidal neurons in association with PART remains unknown. In general, axonal and dendritic swelling are probably suggestive of impaired intraneuronal trafficking. Intracellular sorting and transportation of macro- and micro-molecules between the somata, dendrites, and axons are vital for the anatomical and physiological hemostasis of neurons, which are dependent on complex cellular and molecular interplays that require extensive investigations in the future (Stokin et al., 2005; Kimura and Yanagisawa, 2018; Perdigão et al., 2020). During intraneuronal tangle formation, pTau is disengaged from the microtubules and redistributed from the axon to the soma and dendrites (Hall and Yao, 2000; Spires-Jones et al., 2009). Therefore, dendritic dystrophy might occur as a result of compromised intracellular pTau trafficking. Alternatively, more sophisticated *in vivo* factors and mechanisms might govern the site- and cell-type-specific vulnerability to pathogenesis in various neurodegenerative diseases, including AD. For instance, anatomically, the CA1/subicular region serves as a key neurocircuitry “hub,” integrating the limbic system and cerebral neocortex (Berron et al., 2020). The early involvement of hippocampal and subicular pyramidal neurons in PART, including the dendritic dystrophy identified in the current study, could be related to a high burden of neurotransmission among these “hub” neurons. Such a high activity burden would require dynamic molecular orchestration inside the neuronal somata and processes and, consequently, could affect the involved neurons to be inherently vulnerable to maladaptive failure in maintaining normal intracellular trafficking.

## Conclusion

The present study shows that the apical dendrites of hippocampal and subicular neurons in aged human brains with PART are prone to early dystrophic changes manifested as microscopically overt segmental fusiform swelling. This dendritic malformation occurs mostly at the distal portions or branches of the apical dendrites located in the strata radiatum and stratum lacunosum-moleculare. According to Braak’s classification, stages I to III tauopathy involve pre-tangle and tangle-prone neuronal lesions during AD pathogenesis (Braak and Braak, 1991; Braak et al., 2006). Therefore, our findings indicate that dendritic dystrophy can occur early among limbic projective neurons independent of or prior to senile plaque formation, and this neuritic pathology also develops at the relatively early stages of AD-type tangle pathogenesis.

## Data Availability Statement

The raw data supporting the conclusions of this article will be made available by the authors, without undue reservation.

## Ethics Statement

Written consent for whole body donation for medical education and research was obtained from the donors or next of kin of subjects in compliance with the body/organ donation laws and regulations set by Chinese government. Use of postmodern human brains was approved by the Ethics Committee for Research and Education at Xiangya School of Medicine, in compliance with the Code of Ethics of the World Medical Association (Declaration of Helsinki).

## Author Contributions

ET and X-XY designed the experiments. TT, JJ, Q-LZ, J-QA, and AP contributed to brain banking and tissue processing. TT and Y-BS performed histological experiments and conducted image and data analyses. ET drafted the manuscript. JM and X-XY finalized the manuscript. All the authors read and approved the final manuscript.

## Conflict of Interest

The authors declare that the research was conducted in the absence of any commercial or financial relationships that could be construed as a potential conflict of interest.

## Abbreviations

Aβ: β-amyloid
AD: Alzheimer’s disease
APP: β-amyloid precursor protein
BACE1: β-secretase 1
Col-S: collateral sulcus
DW: de-ionized water
DG: dentate gyrus
DNs: dystrophic neurites
EM: electron microscopy
FG: fusiform gyrus
GCL: granule cell layer
Hi: hilus
HF: hippocampal fissure
ITG: inferior temporal gyrus
ML: molecular layer
NFT: neurofibrillary tangle
Ots: occipito-temporal sulcus
PHF: paired helical filaments
PHG: parahippocampal gyrus
para-S: parasubiculum
PBS: phosphate-buffered saline
pTau: phosphorylated tau
Pre-S: presubiculum
PART: primary age-related tauopathy
Pro-S: prosubiculum
Sorfra: sortilin C-terminal fragments
s.l.m.: stratum lacunosum-moleculare
s.o.: stratum oriens
s.p.: stratum pyramidale
s.r.: stratum radiatum
Sub: subiculum
TC: temporal neocortex
Vps10p: vacuolar protein sorting 10 protein.
